# TWIST1-EP300 Expedites Gastric Cancer Cell Resistance to Apatinib by Activating the Expression of COL1A2

**DOI:** 10.1155/2022/5374262

**Published:** 2022-02-22

**Authors:** Gang Yu, Wanjing Chen, Xianghua Li, Liang Yu, Yanyan Xu, Qiang Ruan, Yawei He, Yong Wang

**Affiliations:** ^1^Department of General Surgery, The Second Hospital of Anhui Medical University, Hefei, 230601 Anhui, China; ^2^Department of Molecular Pathology, Hefei Da'an Medical Laboratory Co., Ltd., Hefei, 230012 Anhui, China

## Abstract

The association between collagen type I alpha (COL1A) and chemoresistance has been verified in cancers. However, the specific role of COL1A2 in gastric cancer (GC) cell resistance to apatinib, a highly selective small-molecule inhibitor of vascular endothelial growth factor receptor 2, has not been investigated before. The purpose of this study was to explore the potential factors associated with COL1A2 regulation on GC cell apatinib resistance *in vitro*. With the aid of the Oncomine database and integrated bioinformatics methods, we identified COL1A2 overexpression in GC and its prognostic value. Mechanistically, the COL1A2 promoter has a distinct H3K27ac modification site and that E1A binding protein p300 (EP300) and twist family bHLH transcription factor 1 (TWIST1) can bind to the COL1A2 promoter, which in turn transcriptionally activated COL1A2 expression. In addition, overexpression of COL1A2 significantly promoted resistance to apatinib in GC cells, but knockdown of EP300 or TWIST1 remarkably inhibited COL1A2 expression and promoted sensitivity of GC cells to apatinib. Our findings demonstrated that the combination of EP300 and TWIST1 has a synergistically regulatory effect on COL1A2 expression, thus contributing to apatinib resistance in GC cells.

## 1. Introduction

In China, gastric cancer (GC) ranks the second place in the incidence and mortality, just behind lung cancer [1]. Patients with locally advanced or metastatic diseases have a somber prognosis, with a median overall survival of 10-12 months, and chemotherapy is the corner stone of treatments [2]. The regimen combining a fluoropyrimidine + a platinum-based agent + an anthracycline or docetaxel is a standard first-line therapy, and second lines of therapy may include irinotecan and taxanes [3]. After failure of second-line treatment, the results of further chemotherapies are disappointing, and no chemotherapeutic agents showed a clear benefit in prolonging survival. Therefore, the discovery of novel and effective treatment options is clinically needed for metastatic GC.

Apatinib mesylate, with a chemical name of N-[4(1-cyano-cyclopentyl)phenyl]-2-(4-pyridylmethyl)amino-3-pyridine carboxamide mesylate, is an orally administered small-molecule inhibitor that targets vascular endothelial growth factor receptor 2 (VEGFR2) [4]. With a favorable side effect profile and improved outcomes, apatinib has demonstrated a considerable potential to enhance therapeutic options in various tumor types [5]. More importantly, apatinib improved the median overall survival of patients with advanced GC after two or more lines of prior chemotherapy of approximately 3 months [6]. Unfortunately, the antiangiogenic strategy has a major practical limitation: resistance inevitably develops [7]. However, the underlying molecular mechanism of apatinib resistance in GC remains largely unclear. Collagen type I (COL1) is the most abundant matrix protein in the cancer stroma, and COL1 could promote tumor progression by facilitating processes, such as cancer cell growth, invasion, and metastasis, and by supporting anticancer drug resistance [8]. Interestingly, COL1A2 was found as a gene with greater than a 50-fold increase in expression in cisplatin-, paclitaxel-, doxorubicin-, topotecan-, vincristine-, and methotrexate-resistant ovarian cancer cells [9]. Consistently, COL1A2 was identified as one of the 21 upregulated genes in 3 datasets between GC tissues and adjacent normal tissues [10]. However, there is little information regarding the regulatory role of COL1A2 in apatinib resistance. Mechanistically, T-box transcription factor 3 was revealed to bind to and activate the COL1A2 promoter in fibrosarcoma and chondrosarcoma [11]. Therefore, we suspected that there is also a transcription factor regulating the COL1A2 expression in GC as well. In the present study, we explored the detailed molecular mechanism of COL1A2 in apatinib-resistant GC. Our study might provide new insights into the chemoresistance of GC at the molecular level and improve the outcomes of GC patients by exploring potential therapeutic targets to overcome apatinib resistance.

## 2. Methods and Materials

### 2.1. Bioinformatics Analysis

Genes with differential expression in Chen Gastric [12], Cho Gastric [13], Cui Gastric [14], Deng Gastric [15], and Wang Gastric [15] datasets were analyzed by cancer vs. normal analysis in the Oncomine database (https://www.oncomine.org/). ChIP-seq data of GC (GSE144385 and GSE162213) were downloaded from the Gene Expression Omnibus (GEO) database (https://www.ncbi.nlm.nih.gov/geo/), analyzed using MACS2, and visualized using IGV software. Subsequently, the expression of COL1A2, EP300, and TWIST1 was queried from TCGA-stomach adenocarcinoma (STAD) database (https://www.cancer.gov/types/stomach).

### 2.2. Cell Culture and Reagents

Human GC cell lines MKN-45 and AGS were purchased from China Center for Type Culture Collection (Wuhan, Hubei, China). The cells were cultured in Roswell Park Memorial Institute- (RPMI-) 1640 containing 10% fetal bovine serum (FBS), 100 U/mL penicillin (15140163, Invitrogen Inc., Carlsbad, CA, USA), and 100 mg/mL streptomycin (31870082, Thermo Fisher Scientific Inc., Waltham, MA, USA) at 37°C in a 5% CO_2_ incubator.

The AGS/MKN-45 cells were treated with apatinib (Hengrui, Lianyungang, Jiangsu, China) at a concentration of 0.001 *μ*M for 48 h, respectively. Then, the medium was replaced with fresh RPMI-1640 medium for subsequent incubation. The adherent cells were further incubated with increasing concentrations of apatinib. Finally, the cells that stably survived at a concentration of 1 *μ*M apatinib were considered AGS or MKN-45 resistant cells (i.e., AGS/R and MKN-45/R).

COL1A2 overexpression plasmids, short hairpin RNAs (shRNAs) targeting COL1A2, TWIST1, and EP300, and their corresponding negative controls (oe-NC or Scramble shRNA) were purchased from GenePharma (Shanghai, China). Overexpression plasmids and oligonucleotides were transfected into the cells using Lipofectamine 2000 reagent (11668030, Invitrogen) for 48 h. The transfected cells were screened using 500 ng/mL puromycin (A1113802, Thermo Fisher).

To further analyze the histone modifications that promote COL1A2 expression, we treated parental AGS and MKN-45 cells with 50 *μ*M C646 (T2452, Tsbiochem, Shanghai, China), a histone acetyl transferase (HAT) EP300 inhibitor, or equal doses of DMSO (D2650, Merck Millipore, Darmstadt, Germany).

### 2.3. Cell Counting Kit-8 (CCK-8)

The AGS/MKN-45 cells were treated with different doses of apatinib (0.001, 0.01, 0.1, and 1 *μ*M) and incubated with 10 *μ*L CCK-8 reagent (BB-4202, BestBio, Shanghai, China) for 2 h. The optical density (OD) value was then measured at 450 nm. The cell viability was calculated according to the following formula: cell viability (%) = [OD(experimental) − OD(blank)]/[OD(control) − OD(blank)] × 100%. Median inhibition concentration (IC50) was calculated by using GraphPad Prism.

### 2.4. RNA Extraction and Real-Time Quantitative Polymerase Chain Reaction (RT-qPCR)

The mRNA expression of target genes in GC tissues was detected by RT-qPCR. Total RNA was first extracted from tissues using TRIzol reagent (15596026, Thermo Fisher) and reverse transcribed into cDNA using the M-MuLV kit (42°C for 60 min; 70°C for 15 min, maintained at 16°C) according to the manufacturer's instructions (B532435-0010, Sangon Biotech, Shanghai, China). The SYBR Green PCR kit (C0006, Tsbiochem) and CFX-Connect 96 instruments were utilized. Glyceraldehyde-3-phosphate dehydrogenase (GAPDH) was used as an interior parameter. The primers used in this research are listed in Table 1.

### 2.5. Western Blot

Protein samples (30 *μ*g) from the cells were lysed in radioimmunoprecipitation assay lysis buffer (P0013K, Beyotime Biotechnology, Shanghai, China) and quantified using the bicinchoninic acid assay protein assay kit (P0009, Beyotime). Then, equal amounts of protein samples (30 *μ*g) were separated by electrophoresis on 10% sodium dodecyl sulfate-polyacrylamide gels and transferred to polyvinylidene difluoride membranes. Subsequently, the membranes were blocked with 5% (*w*/*v*) skim milk for 2 h, followed by an overnight incubation with antibodies to COL1A2 (1 : 1000, GTX102996, GeneTex, Inc., Alton Pkwy Irvine, CA, USA), TWIST1 (1 : 1000, GTX60776, GeneTex), and EP300 (1 : 1000, ab275378, Abcam, Cambridge, UK) and a 2 h incubation with goat anti-mouse secondary antibody (1 : 15,000, ab205719, Abcam) or goat anti-rabbit IgG (1 : 15,000, ab6721, Abcam) at room temperature. Bands were visualized using a chemiluminescent substrate kit (P0018FS, Beyotime). GAPDH (1 : 1000, ab8245, Abcam) was used as an internal control.

### 2.6. Colony Formation Assay

The AGS/MKN-45 cells were dispersed into a single cell suspension and seeded into 6-well plates (400 cells/2 mL/well) and gently rotated to distribute the cells evenly. After maintaining at 37°C and 5% CO_2_ for 24 h, the cells were incubated with RPMI-1640 containing 10% FBS and 1 *μ*M apatinib for 2 weeks. When colonies were visible in the Petri dish with the naked eyes, the cells were carefully washed with phosphate-buffered saline (PBS) twice and fixed with pure methanol for 20 min. Finally, we stained the cells with 0.5% crystal violet solution and counted the number of colonies formed under an inverted light microscope (Nikon Corporation, Tokyo, Japan).

### 2.7. Flow Cytometry

After 48 h of transfection, AGS/MKN-45 cells (5 × 10^5^ cells/mL) were trypsinized and then collected for analysis. The cells were stained in the dark for 30 min by applying annexin V-fluorescein isothiocyanate (FITC) and propidium iodide (PI) according to the instructions of the Apoptosis Detection Kit (C1062S, Beyotime). Finally, apoptosis rates were detected within 1 h after staining using an ACEABIO NovoCyte flow cytometer (ACEA Biosciences Inc., San Diego, CA, USA).

### 2.8. Terminal Deoxynucleotidyl Transferase- (TdT-) Mediated 2′-Deoxyuridine 5′-Triphosphate (dUTP) Nick End Labeling (TUNEL)

The transfected AGS/MKN-45 cells were fixed with 4% paraformaldehyde for 20 min at 4°C. Apoptosis was detected at 37°C for 1 h using the TUNEL Apoptosis Assay Kit (C10617, Invitrogen) according to the manufacturer's instructions. Subsequently, the cells were incubated with diaminobenzene for 10 min and counterstained with hematoxylin (C0107, Beyotime) for 30 s at room temperature. Apoptosis of TUNEL-positive cells was observed under a light microscope (magnification, ×200; Olympus Optical Co., Ltd., Tokyo, Japan), and cells were counted in five randomly selected microscopic fields.

### 2.9. Chromatin Immunoprecipitation (ChIP)

ChIP was performed using the Magna ChIP™ A/G One-Day ChIP Kit (Catalog #17-10085; Merck) according to the manufacturer's instructions. Cross-linking of chromatin was achieved by using 1% formaldehyde for 10 min at 37°C and neutralizing with glycine for 5 min at room temperature. All GC cells were washed with cold 1 mL PBS + proteinase inhibitor (1 mM phenylmethanesulfonyl fluoride, 1 mg peptidase, and 1 mg gastrase inhibitor A). The cells were centrifuged at 716 × *g* for 5 min at 4°C and disrupted using sodium dodecyl sulfate (SDS) lysis buffer (1% SDS, 10 mM ethylenediaminetetraacetic acid and 50 mM Tris-Hcl pH = 8.0). Subsequent sonication at 150 Hz was performed with four sets of 10 s pulse shears on ice using a high-intensity ultrasonic processor (Cole-Parmer). Equal amounts of chromatin were immunoprecipitated with antibodies targeting TWIST1 (1 : 500, #GTX60776, GeneTex) or H3K27ac (1 : 500, #GTX50903, GeneTex) overnight at 4°C with anti-mouse IgG (1 : 500, ab18413; Abcam) as an isotype control. The total chromatin was used as input. Immunoprecipitation products were collected after incubation with magnetic beads. Beads were washed using a magnetic separation rack, and bound chromatin was eluted in ChIP elution buffer with a proteinase K mixer according to the manufacturer's instructions.

### 2.10. Dual-Luciferase Reporter Assay

The targeting relationship between TWIST1 and COL1A2 was verified using a dual-luciferase reporter gene assay. The pGL3-COL1A2 wild-type (WT) luciferase reporter gene vector was designed and synthesized based on the binding sequence of the COL1A2 promoter region to TWIST1. The pGL3-COL1A2 plasmid was then cotransfected with TWIST1 overexpression vectors at different doses into HEK293T cells (ACS-4500, American Type Culture Collection, Manassas, VA, USA). The cells were lysed after 48 h. The supernatant was obtained by centrifugation at 12,000 rpm for 1 min. Luciferase activities were measured using the Dual-Luciferase® Reporter Assay System (E1910, Promega Corp., Madison, WI, USA), with renilla luciferase as an internal control.

### 2.11. Double-Labeled Immunofluorescence

The cells were treated with 4% paraformaldehyde (E672002, Sangon) and 0.5% Triton X-100 (Sangon). The cells were treated with TWIST1 (1 : 500, #GTX60776, GeneTex) and EP300 (1 : 100, ab275378, Abcam) and stained with FITC-conjugated goat anti-rabbit antibody (ab6717, Abcam). The cells were then denatured with 2 M hydrochloric acid at 37°C for 10 min and then stained with Alexa Fluor 647-conjugated goat anti-mouse antibody (1 : 500, ab150083, Abcam). The cells were mounted in medium containing an antifluorescent quenching agent, and the nucleus was stained with 0.25 *μ*g/mL 4′,6-diamidino-2-phenylindole (C1005, Beyotime Biotech). Then, the fluorescent images were captured with a laser scanning confocal microscope (Leica, Frankfurt, Germany) equipped with the appropriate FITC and Texas Red filters.

### 2.12. Data Analysis

In this study, data were analyzed with SPSS software (version 21.0, IBM Corp., Armonk, NY, USA). All experiments were repeated at least three times, and the results were showed as the mean ± standard deviation (SD). Unpaired *t*-test and one-way or two-way analysis of variance with Tukey's correction were applied to analyze the statistics. When *p* value was less than 0.05, the differences between each group were considered significant.

## 3. Results

### 3.1. COL1A2 Is Highly Expressed in GC Patients and Associated with Poor Prognosis and Apatinib Resistance

**Figure 1:**
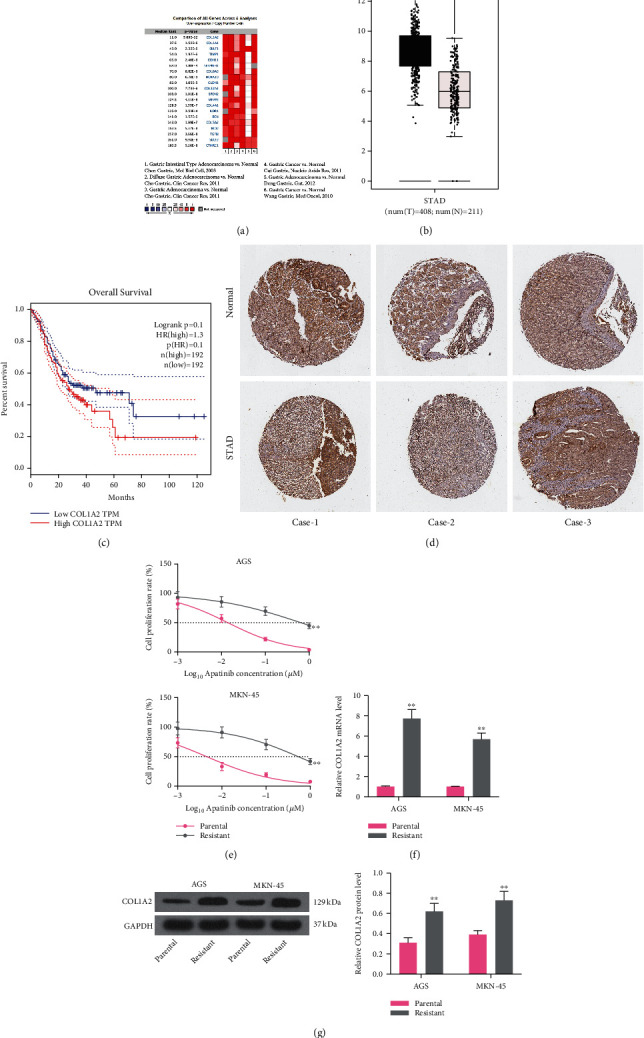
COL1A2 is highly expressed in GC patients and is associated with poor prognosis and apatinib resistance. (a) Genes with differential expression in the Chen Gastric, Cho Gastric, Cui Gastric, Deng Gastric, and Wang Gastric datasets analyzed by cancer vs. normal analysis in the Oncomine database. (b) Analysis of COL1A2 expression in normal tissues and GC tissues in TCGA-STAD database. (c) Kaplan-Meier analysis of the relationship between COL1A2 and survival in patients with GC in TCGA-STAD database. (d) Analysis of COL1A2 staining intensity in GC tissues versus normal gastric tissues measured using the HPA website. (e) IC50 values of parental and drug-resistant cells examined using the CCK-8 assay. (f) Detection of COL1A2 mRNA expression in parental and drug-resistant cells by RT-qPCR. (g) Detection of COL1A2 protein expression in parental and drug-resistant cells by western blot. Data indicate means ± SD of three biological replicates. Two-way ANOVA and Tukey's multiple comparison test; ^∗∗^*p* < 0.01 (vs. parental cells).

In order to explore the pathogenesis and treatment of GC, we first analyzed the differentially expressed genes in Chen Gastric, Cho Gastric, Cui Gastric, Deng Gastric, and Wang Gastric datasets by cancer vs. normal analysis in the Oncomine database (Figure 1(a)). We screened out that COL1A2 was the gene with the most pronounced expression differences. We further found in TCGA-STAD database that the expression of COL1A2 was significantly increased in GC tissues and was associated with poor prognosis of patients (Figures 1(b) and 1(c)). Subsequently, we further observed from the HPA website (https://www.proteinatlas.org/) that the intensity of COL1A2 staining was much stronger in GC tissues than in paracancerous tissues (Figure 1(d)).

Subsequently, we exposed AGS and MKN-45 cells to graded concentrations of apatinib for the construction of cell lines resistant to apatinib. After 6 months of continuous culture, we examined the IC50 values of parental and resistant cells to apatinib using the CCK-8. We found that the sensitivity of resistant cells to apatinib was significantly reduced (*p* < 0.01) (Figure 1(e)), indicating that the resistant cell line was successfully constructed. In addition, a significant increase in COL1A2 expression at mRNA and protein levels was observed in drug-resistant cells (all *p* < 0.01) (Figures 1(f) and 1(g)).

### 3.2. TWIST1 Is Positively Correlated with COL1A2 and Transcriptionally Activates COL1A2

**Figure 2:**
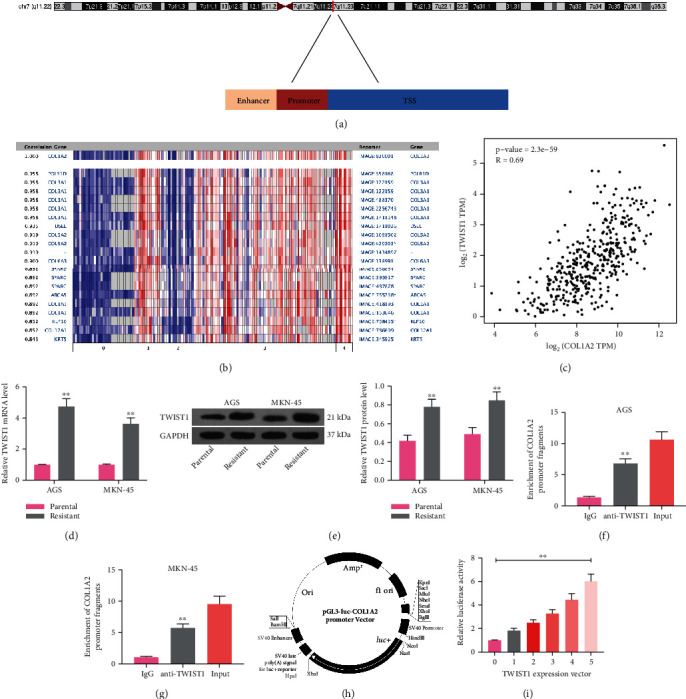
TWIST1 is positively correlated with COL1A2 and transcriptionally activates COL1A2. (a) The promoter of COL1A2 was queried in the Ensembl website at Chr7:94394582~94394928. (b) Analysis of the top 30 genes with the highest association with COL1A2 in the Chen Gastric, Cho Gastric, Cui Gastric, Deng Gastric, and Wang Gastric datasets in the Oncomine website. (c) Spearman correlation analysis of TWIST1 with COL1A2 in TCGA-STAD database. (d) The mRNA expression of TWIST1 in parental and drug-resistant cells by RT-qPCR. (e) The protein expression of TWIST1 in parental and drug-resistant cells by western blot. (f, g) The binding of TWIST1 to the COL1A2 promoter in parental cells examined using ChIP-qPCR. (h) Schematic diagram of the pGL3-Luc luciferase reporter vector containing the COL1A2 promoter. (i) The luciferase reporter vector was cotransfected into 293T cells together with different doses of TWIST1 overexpression plasmids to detect changes in luciferase activity in the cells. Data indicate means ± SD of three biological replicates. Two-way ANOVA and Tukey's multiple comparison test; ^∗∗^*p* < 0.01 (vs. parental cells, IgG, or 0).

To explore the upstream regulatory mechanism of COL1A2, we conducted a series of prediction and assays. Firstly, we queried the promoter of COL1A2 located in Chr7:94394582~94394928 (Figure 2(a)) from the Ensembl website. Subsequently, we further analyzed the top 30 genes with the highest association with COL1A2 in the datasets of Chen Gastric, Cho Gastric, Cui Gastric, Deng Gastric, and Wang Gastric by coexpression analysis in the Oncomine website. We screened out TWIST1 (Figure 2(b)) from them. Moreover, in TCGA-STAD database, a positive correlation between TWIST1 and COL1A2 was identified using Spearman's correlation analysis (Figure 2(c)). Also, we found that the expression of TWIST1 in drug-resistant cells was also significantly higher than that in parental cells (all *p* < 0.01) (Figures 2(d) and 2(e)).

Thus, we performed ChIP-qPCR using antibody against TWIST1 in order to verify whether TWIST1 can bind to the promoter of COL1A2 as a transcription factor, and we observed that anti-TWIST1 can enrich more COL1A2 promoter fragment relative to IgG (all *p* < 0.01) (Figures 2(f) and 2(g)). To further verify that TWIST1 can transcriptionally activate COL1A2 expression, we designed pGL3-Luc luciferase reporter vector containing COL1A2 promoter sequence (Figure 2(h)) and cotransfected it into 293T cells together with different concentrations of TWIST1 overexpression plasmids. It was observed that as the concentration of TWIST1 increased, the luciferase activity in the cells had a significant increase (*p* < 0.01) (Figure 2(i)). The above results indicated that TWIST1 can transcriptionally activate the expression of COL1A2.

### 3.3. The COL1A2 Promoter Has a Distinct Enhancer-Like Signature

**Figure 3:**
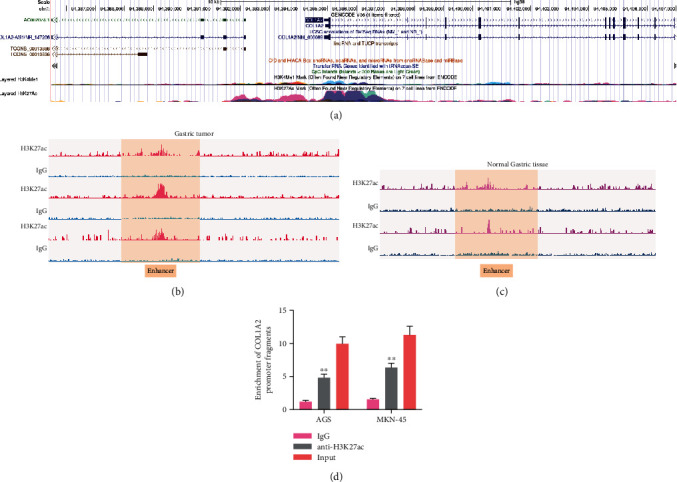
The COL1A2 promoter is significantly regulated by an enhancer-like signature. (a) COL1A2 promoter modification level searched by UCSC browser. (b, c) ChIP-seq data of H3K27ac in GC and normal gastric tissues were downloaded from the GEO database and analyzed for enrichment in the COL1A2 promoter. (d) ChIP-qPCR analysis of COL1A2 promoter histone H3K27ac modification levels in AGS and MKN-45 cells. Data indicate means ± SD of three biological replicates. Two-way ANOVA and Tukey's multiple comparison test; ^∗∗^*p* < 0.01 (vs. IgG).

We further found a significant H3K27ac modification near the promoter of COL1A2 through the UCSC browser website (http://genome.ucsc.edu/index.html) (Figure 3(a)). Subsequently, we downloaded the ChIP-seq data of H3K27ac from the GEO database for GC tissues with corresponding paracancerous tissues (GSE144385 and GSE162213). The promoter of COL1A2 had higher H3K27ac modification in GC tissues (Figures 3(b) and 3(c)). Thus, we further employed ChIP-qPCR analysis on AGS and MKN-45 cells, and we found that the use of anti-H3K27ac antibody enriched more promoter sequences of COL1A2 (*p* < 0.01) (Figure 3(d)).

### 3.4. EP300 Promotes COL1A2 Expression through a H3K27ac-Dependent Manner

**Figure 4:**
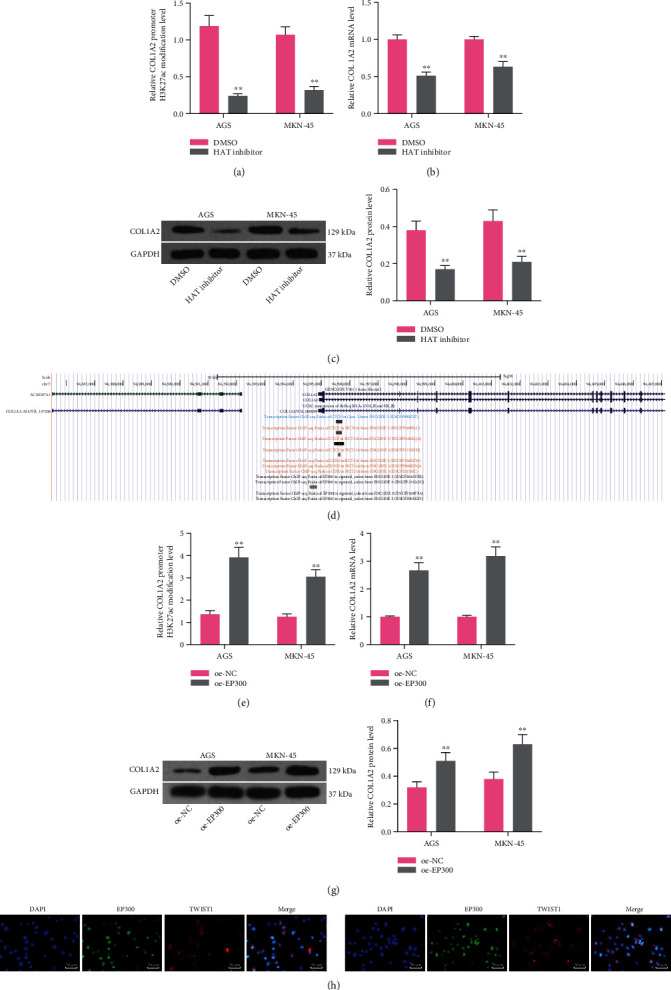
EP300 promotes COL1A2 expression through an H3K27ac-dependent manner. GC cells were treated with HAT inhibitor or DMSO. (a) ChIP-qPCR analysis of the H3K27ac modification level of COL1A2 promoter. (b) The mRNA expression of COL1A2 in parental cells by RT-qPCR. (c) The protein expression of COL1A2 in parental cells by western blot. (d) UCSC browser analysis of the binding of EP300 to the promoter of COL1A2. GC cells were transfected with oe-NC or oe-EP300. (e) ChIP-qPCR analysis of the H3K27ac modification level of COL1A2 promoter. (f) The mRNA expression of COL1A2 in parental cells by RT-qPCR. (g) The protein expression of COL1A2 in parental cells by western blot. (h) Colocalization of TWIST1 with EP300 in AGS and MKN-45 cells by double-labeled immunofluorescence staining. Data indicate means ± SD of three biological replicates. Two-way ANOVA and Tukey's multiple comparison test; ^∗∗^*p* < 0.01 (vs. DMSO or oe-NC).

To further analyze the histone-modifying enzymes that promote COL1A2 expression, we first treated AGS and MKN-45 cells with a HAT inhibitor, and we found that the level of H3K27ac modification of the COL1A2 promoter was significantly reduced in the cells after the use of HAT inhibitor (*p* < 0.01), and the mRNA and protein expression of COL1A2 was similarly reduced (all *p* < 0.01) (Figures 4(a)–4(c)). We further observed in the UCSC website that EP300 had a binding relationship with the promoter of COL1A2 (Figure 4(d)).

To clarify that EP300 promoted H3K27ac modification of the COL1A2 promoter and thus COL1A2 expression, we transfected EP300 overexpression plasmids into AGS or MKN-45 cells. After that, a significant increase in the level of H3K27ac modification was observed (both *p* < 0.01) (Figure 4(e)). Moreover, the enhanced mRNA and protein expression of COL1A2 was observed as well (all *p* < 0.01) (Figures 4(f) and 4(g)). Subsequently, we further found that TWIST1 colocalized with EP300 in AGS and MKN-45 cells by double-labeled immunofluorescence (Figure 4(h)). This result suggests that during the transcriptional activation of COL1A2 by TWIST1, EP300 was recruited near the promoter of COL1A2, which in turn promotes H3K27ac modification, leading to the overexpression of COL1A2.

### 3.5. Overexpression of COL1A2 Promotes Drug Resistance to Apatinib in Parental GC Cells

**Figure 5:**
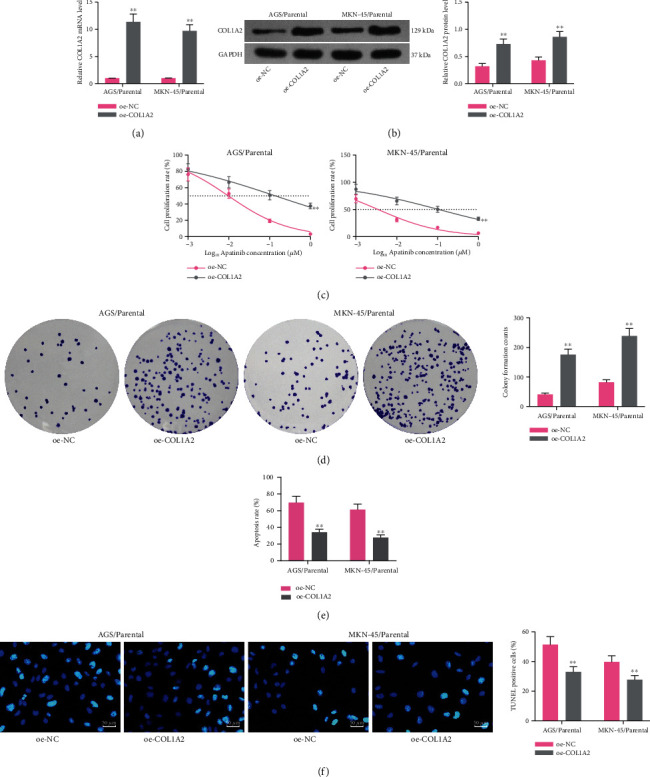
Overexpression of COL1A2 promotes resistance of parental cells to apatinib. GC parental cells were transfected with oe-NC or oe-COL1A2. (a) The mRNA expression of COL1A2 in parental cells by RT-qPCR. (b) The protein expression of COL1A2 in parental cells by western blot. (c) The IC50 values of parental cells by CCK-8 assay. (d) The number of colonies formed by parental MKN-45 and AGS cells treated with 1 *μ*M apatinib. (e) Flow cytometry analysis of the proportion of apoptotic cells in parental MKN-45 and AGS cells. (f) TUNEL analysis of the number of apoptotic bodies in parental MKN-45 and AGS cells. Data indicate means ± SD of three biological replicates. Two-way ANOVA and Tukey's multiple comparison test; ^∗∗^*p* < 0.01 (vs. oe-NC).

Based on the aforementioned speculation that COL1A2 was significantly correlated with apatinib resistance in GC cells, we overexpressed COL1A2 in parental AGS and MKN-45 cells. RT-qPCR and western blot were utilized to verify the transfection efficiency (all *p* < 0.01) (Figures 5(a) and 5(b)). Subsequently, we examined the IC50 value of parental GC cells using the CCK-8 assays. The repressive effect of apatinib on GC cell proliferation was significantly reduced after overexpression of COL1A2 (both *p* < 0.01) (Figure 5(c)). Similarly, a significant increase in the number of colonies formed by cells after treatment with 1 *μ*M apatinib (both *p* < 0.01) (Figure 5(d)) and a significant decline in the number of apoptotic cells (all *p* < 0.01) (Figures 5(e) and 5(f)) were observed.

### 3.6. Knockdown of COL1A2 Sensitizes Drug-Resistant Cells to Apatinib

**Figure 6:**
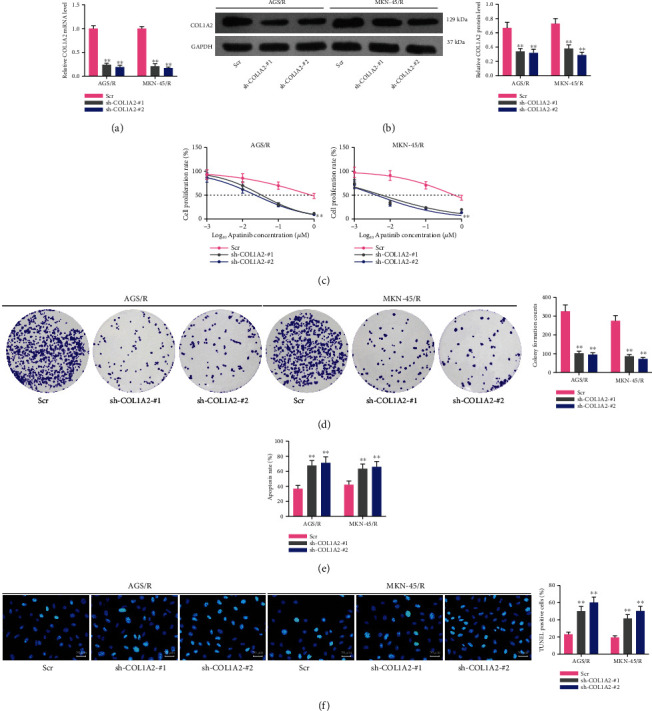
Knockdown of COL1A2 inhibits the sensitivity of drug-resistant cells to apatinib. GC-resistant cells were transfected with Scr or sh-COL1A2 #1 or #2. (a) The mRNA expression of COL1A2 in resistant cells by RT-qPCR. (b) The protein expression of COL1A2 in resistant cells by western blot. (c) The IC50 values of resistant cells by CCK-8 assay. (d) The number of colonies formed by resistant MKN-45 and AGS cells treated with 1 *μ*M apatinib. (e) Flow cytometry analysis of the proportion of apoptotic cells in resistant MKN-45 and AGS cells. (f) TUNEL analysis of the number of apoptotic bodies in resistant MKN-45 and AGS cells. Data indicate means ± SD of three biological replicates. Two-way ANOVA and Tukey's multiple comparison test; ^∗∗^*p* < 0.01 (vs. Scr).

In Figure 5, we found that overexpression of COL1A2 significantly promoted the resistance of parental GC cells to apatinib. To further verify the effect of COL1A2 on apatinib resistance, we transfected shRNAs targeting COL1A2 into resistant cells and confirmed the knockdown efficiency in cells by RT-qPCR with western blot (all *p* < 0.01) (Figures 6(a) and 6(b)). Subsequently, we further determined the changes in the sensitivity of drug-resistant cells to apatinib after knockdown of COL1A2 by CCK-8. The sensitivity of MKN-45/R and AGS/R cells to apatinib was significantly increased in the presence of shRNA-COL1A2 (both *p* < 0.01) (Figure 6(c)), and the inhibiting effects of cell growth by 1 *μ*M apatinib were also significantly enhanced (both *p* < 0.01) (Figure 6(d)). In addition, the number of cell death was elevated after treatment (all *p* < 0.01) (Figures 6(e) and 6(f)).

### 3.7. Knockdown of TWIST1 or EP300 Reduces Resistance of Parental Cells Overexpressing COL1A2 to Apatinib

**Figure 7:**
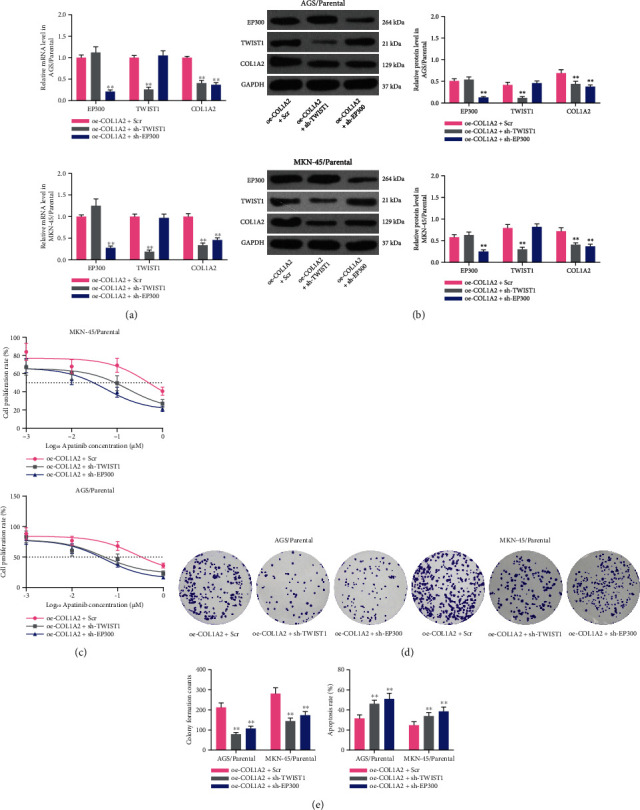
Knockdown of TWIST1 or EP300 reduces resistance to apatinib in parental cells overexpressing COL1A2. GC parental cells transfected with oe-COL1A2 were further transfected with sh-TWIST1 or sh-EP300. (a) The mRNA expression of TWIST1, EP300, and COL1A2 in parental cells by RT-qPCR. (b) The protein expression of TWIST1, EP300, and COL1A2 in parental cells by western blot. (c) The IC50 values of parental cells by CCK-8 assay. (d) The number of colonies formed by parental MKN-45 and AGS cells treated with 1 *μ*M apatinib. (e) Flow cytometry analysis of the proportion of apoptotic cells in parental MKN-45 and AGS cells. Data indicate means ± SD of three biological replicates. Two-way ANOVA and Tukey's multiple comparison test; ^∗∗^*p* < 0.01 (vs. oe − COL1A2 + Scr).

To explore the effect of TWIST1 or EP300 on apatinib in GC cells, we transfected shRNAs targeting TWIST1 or EP300 into cells overexpressing COL1A2 and verified the transfection efficiency using RT-qPCR and western blot (all *p* < 0.01) (Figures 7(a) and 7(b)). We observed a significant increase in the sensitivity of MKN-45 or AGS cells to apatinib (both *p* < 0.01) (Figure 7(c)). Knockdown of TWIST1 or EP300 further strengthened the effects of apatinib on GC cell growth and death (all *p* < 0.01) (Figures 7(d) and 7(e)).

## 4. Discussion

Apatinib has been revealed to have anticancer effects through stimulation of apoptosis, suppression of cell proliferation, and induction of the effects of conventional chemotherapy agents [16–18]. Moreover, it is expected to have a wider application when drug resistance is overcome [5]. Drug resistance is particularly important for patients who proceed to second-line or above chemotherapy since there are no further chemodrugs available once resistance to apatinib occurs, and antiangiogenic agent resistance is suggested to be mainly associated with activation of cell adhesion-related or growth-related signaling pathways [19]. This study showed that COL1A2 potentiated the resistance of GC cell to apatinib *in vitro*. The mechanism involved in the process was that COL1A2 expression was elevated by the transcription factor TWIST1, and EP300 was recruited near the promoter of COL1A2 to increase its H3K27ac modification, leading to COL1A2 overexpression and apatinib resistance in GC cells.

In our study, we first verified the significant overexpression of COL1A2 in GC using five relevant datasets and clarified its association with the somber prognosis of GC patients. To decipher the detailed mechanism for the overexpression of COL1A2 in GC, integrated bioinformatics tools were utilized, which exhibited that TWIST1 showed a significant positive correlation with COL1A2. TWIST1 is a basic helix-loop-helix transcription factor which plays pivotal parts in manifold stages of embryonic development and considerably results in tumor initiation, primary tumor growth, and even tumor metastasis [20]. Interestingly, TWIST1 transcriptionally upregulated AKT2 in breast cancer cells, leading to increased migration, invasion, and resistance to paclitaxel [21]. Kim et al. showed using ChIP that UV irradiation reduced the local recruitment of histone H3 acetylation as well as EP300 to the binding site (-1406/-1393) in the COL1A2 promoter in human dermal fibroblasts [22]. Our ChIP-seq data also revealed the distinct H3K27ac modification in the COL1A2 promoter in GC tissues, which was validated using ChIP-qPCR. Moreover, the interaction between EP300 and TWIST1 has been attested by Hamamori et al. [23]. Therefore, we believed that the overexpression of COL1A2 in GC was caused by the coordination between EP300 and TWIST1. A similar mechanism was observed in multiple myeloma cells as well where SP1/EP300 complex promoted proliferation of cancer cells through modulating the transcription of IQGAP1 [24]. Furthermore, Asano and Trojanowska reported that Fli1 recruited HDAC1/EP300 to the COL1A2 promoter and suppressed the expression of the COL1A2 through histone deacetylation in human dermal fibroblasts [25]. These discordant results may be due to the influence of HDAC1, a histone deacetylase.

Later, we conducted gain- and loss-of-function assays to validate their roles in GC cell resistance to apatinib. The interaction between COL1 and cisplatin resistance has been indicated by Rekowski et al. in ovarian cancer [26]. Also, Kyoto Encyclopedia of Genes and Genomes pathway enrichment analysis revealed that COL1A1 regulated carboplatin resistance of ovarian cancer cells through the “ECM-receptor interaction” and “focal adhesion” pathways [27]. In addition, pcCOL1A2 was found to reverse the effects of miR-29a-3p on GC cell proliferation and apoptosis [28]. In the present study, overexpression of COL1A2 effectively enhanced the parental GC cell resistance to apatinib, as evidenced by enhanced cell growth and repressed apoptosis. As for TWIST1, TWIST1 was upregulated in various malignances and has a positive correlation with poor prognosis [29, 30]. In addition, TWIST1 increased (about 2-fold) the ability of thyroid cancer cells to migrate into collagen I matrix, whereas TWIST1 knockdown in CAL62 cells reduced cell migration into collagen I matrix [31]. EP300 (also known as p300 and KAT3B) is a large gene spanning about 90 kilobases on chromosome 22q13.2; it encodes a 2414 amino acid protein with a molecular weight of about 260 kDa [32]. EP300 knockdown abolished the cancer stem cell phenotype by reducing sphere formation capacity *in vitro* as well as tumor formation in a xenograft mouse model in triple-negative breast cancer [33]. More relevantly, Furlan et al. reported that EP300/CREBBP inhibitors mediate the MYC/ribosomal protein axis in enzalutamide-resistant cells and might have promising therapeutic implications in prostate cancer [34].

## 5. Conclusion

Collectively, TWIST1 synergizes with EP300 to enhance COL1A2 expression. COL1A2 could induce the apatinib resistance in GC. In addition, TWIST1 or EP300 depletion could sensitize GC cells to apatinib by blocking COL1A2 expression. However, due to the lack of clinical samples, future validation is needed in both human and animal tissues.

## Figures and Tables

**Table 1:** The primers used in this work.

Symbol	Forward (5′-3′)	Reverse (5′-3′)
COL1A2	CCTGGTGCTAAAGGAGAAAGAGG	ATCACCACGACTTCCAGCAGGA
EP300	GATGACCCTTCCCAGCCTCAAA	GCCAGATGATCTCATGGTGAAGG
TWIST1	GCCAGGTACATCGACTTCCTCT	TCCATCCTCCAGACCGAGAAGG
GAPDH	GTCTCCTCTGACTTCAACAGCG	ACCACCCTGTTGCTGTAGCCAA

Note: COL1A2: collagen type I alpha 2 chain; EP300: E1A binding protein p300; TWIST1: twist family bHLH transcription factor 1; GAPDH: glyceraldehyde-3-phosphate dehydrogenase.

## Data Availability

All the data generated or analyzed during this study are included in this published article.
